# Supplementary material for: The sleep-wake distribution contributes to the peripheral rhythms in PERIOD-2
Source: eLife. 2021 Dec 13;10:e69773. doi: 10.7554/eLife.69773 (PMC8798053; doi:10.7554/eLife.69773)
Supplement: Supplementary file 1. [file elife-69773-supp1.docx]

Overall sequence of the construct:

>pCV100_rLV(no_cloned_UTR)

TTCGAAATTTAAATCATCATCAATAATATACCTTATTTTGGATTGAAGCCAATATGATAATGAGGGGGTGGAGTTTGTGACGTGGCGCGGGGCGTGGGAACGGGGCGGGTGACGTAGTAGTGTGGCGGAAGTGTGATGTTGCAAGTGTGGCGGAACACATGTAAGCGACGGATGTGGCAAAAGTGACGTTTTTGGTGTGCGCCGGTGTACACAGGAAGTGACAATTTTCGCGCGGTTTTAGGCGGATGTTGTAGTAAATTTGGGCGTAACCGAGTAAGATTTGGCCATTTTCGCGGGAAAACTGAATAAGAGGAAGTGAAATCTGAATAATTTTGTGTTACTCATAGCGCGTAATATTTGTCTAGGGCCGCGGGGACTTTGACCGTTTACGTGGAGACTCGCCCAGGTGTTTTTCTCAGGTGTTTTCCGCGTTCCGGGTCAAAGTTGGCGTTTTGATGGCGTCCCTTAATTAACAGGGCCTGCCGCCCCGACGTTGGCTGCGAGCCCTGGGCCTTCACCCGAACTTGGGGGGTGGGGTGGGGAAAAGGAAGAAACGCGGGCGTATTGGCCCCAATGGGGTCTCGGTGGGGTATCGACAGAGTGCCAGCCCTGGGACCGAACCCCGCGTTTATGAACAAACGACCCAACACCGTGCGTTTTATTCTGTCTTTTTATTGCCGTCATAGCGCGGGTTCCTTCCGGTATTGTCTCCTTCCGTGTTTCAGTTAGCCTCCCCCCTCGAGCATATGGCGGCCGCTCGCGACTGCAGTTACACGGCGATCTTGCCGCCCTTCTTGGCCTTAATGAGAATCTCGCGGATCTTGCGGGCGTCCAACTTGCCGGTCAGTCCTTTAGGCACCTCGTCCACGAACACAACACCACCGCGCAGCTTCTTGGCGGTTGTAACCTGGCTGGCCACATAGTCCACGATCTCCTTCTCGGTCATGGTTTTACCGTGTTCCAGCACGACGACTGCGGCGGGCAGCTCGCCGGCATCGTCGTCGGGCAGGCCGGCGACCCCGGCGTCGAAGATGTTGGGGTGTTGCAGCAGGATGCTCTCCAGTTCGGCTGGGGCTACCTGGTAGCCCTTGTATTTGATCAGGCTCTTCAGCCGGTCCACGATGAAGAAGTGCTCGTCCTCGTCCCAGTAGGCGATGTCGCCGCTGTGCAGCCAGCCGTCCTTGTCGATGAGAGCGTTTGTAGCCTCGGGGTTGTTAACGTAGCCGCTCATGATCATGGGGCCACGGACGCACAGCTCGCCGCGCTGGTTCACACCCAGTGTCTTACCGGTGTCCAAGTCCACCACCTTAGCCTCGAAGAAGGGCACCACCTTGCCTACTGCGCCAGGCTTGTCGTCCCCTTCGGGGGTGATCAGAATGGCGCTGGTTGTTTCTGTCAGGCCGTAGCCCTGGCGGATGCCTGGTAGGTGGAAGCGTTTGGCCACGGCCTCACCTACCTCCTTGCTGAGCGGCGCCCCGCCGCTGGCGATCTCGTGCAAGTTGCTTAGGTCGTACTTGTCGATGAGAGTGCTCTTAGCGAAGAAGCTAAATAGTGTGGGCACCAGCAGGGCAGATTGAATCTTATAGTCTTGCAAGCTGCGCAAGAATAGCTCCTCCTCGAAGCGGTACATGAGCACGACCCGAAAGCCGCAGATCAAGTAGCCCAGCGTGGTGAACATGCCGAAGCCGTGGTGAAATGGCACCACGCTGAGGATAGCGGTGTCGGGGATGATCTGGTTGCCGAAGATGGGGTCGCGGGCATGACTGAATCGGACACAAGCGGTGCGGTGCGGTAGGGCTACGCCCTTGGGCAATCCGGTACTGCCACTACTGTTCATGATCAGGGCGATGGTTTTGTCCCGGTCGAAGCTCTCGGGCACGAAGTCGTACTCGTTGAAGCCGGGTGGCAAATGGGAAGTCACGAAGGTGTACATGCTTTGGAAGCCCTGGTAGTCGGTCTTGCTATCCATGATGATGATCTTTTGTATGATCGGTAGCTTCTTTTGCACGTTGAGGATCTTTTGCAGCCCTTTCTTGCTCACGAATACGACGGTGGGCTGGCTGATGCCCATGCTGTTCAGCAGCTCGCGCTCGTTGTAGATGTCGTTAGCTGGGGCCACAGCCACACCGATGAACAGGGCACCCAACACGGGCATGAAGAACTGCAAGCTATTCTCGCTGCACACCACGATCCGATGGTTTGTATTCAGCCCATAGCGCTTCATAGCTTCTGCCAGCCGAACGCTCATCTCGAAGTACTCGGCGTAGGTAATGTCCACCTCGATATGTGCGTCGGTAAAGGCGATGGTGCCGGGCACCAGGGCGTAGCGCTTCATGGCTTTGTGCAGCTGCTCGCCGGCGGTCCCGTCTTCGAGTGGGTAGAATGGCGCTGGGCCCTTCTTAATGTTTTTGGCATCTTCCATggtggCACGCGTggatccttcgagaagcttgatatcgaattcccacggggttggggttgcgccttttccaaggcagccctgggtttgcgcagggacgcggctgctctgggcgtggttccgggaaacgcagcggcgccgaccctgggtctcgcacattcttcacgtccgttcgcagcgtcacccggatcttcgccgctacccttgtgggccccccggcgacgcttcctgctccgcccctaagtcgggaaggttccttgcggttcgcggcgtgccggacgtgacaaacggaagccgcacgtctcactagtaccctcgcagacggacagcgccagggagcaatggcagcgcgccgaccgcgatgggctgtggccaatagcggctgctcagcggggcgcgccgagagcagcggccgggaaggggcggtgcgggaggcggggtgtggggcggtagtgtgggccctgttcctgcccgcgcggtgttccgcattctgcaagcctccggagcgcacgtcggcagtcggctccctcgttgaccgaatcaccgacctctctccccaGctgtatttACGCGTgccaccATGACTTCGAAAGTTTATGATCCAGAACAAAGGAAACGGATGATAACTGGTCCGCAGTGGTGGGCCAGATGTAAACAAATGAATGTTCTTGATTCATTTATTAATTATTATGATTCAGAAAAACATGCAGAAAATGCTGTTATTTTTTTACATGGTAACGCGGCCTCTTCTTATTTATGGCGACATGTTGTGCCACATATTGAGCCAGTAGCGCGGTGTATTATACCAGACCTTATTGGTATGGGCAAATCAGGCAAATCTGGTAATGGTTCTTATAGGTTACTTGATCATTACAAATATCTTACTGCATGGTTTGAACTTCTTAATTTACCAAAGAAGATCATTTTTGTCGGCCATGATTGGGGTGCTTGTTTGGCATTTCATTATAGCTATGAGCATCAAGATAAGATCAAAGCAATAGTTCACGCTGAAAGTGTAGTAGATGTGATTGAATCATGGGATGAATGGCCTGATATTGAAGAAGATATTGCGTTGATCAAATCTGAAGAAGGAGAAAAAATGGTTTTGGAGAATAACTTCTTCGTGGAAACCATGTTGCCATCAAAAATCATGAGAAAGTTAGAACCAGAAGAATTTGCAGCATATCTTGAACCATTCAAAGAGAAAGGTGAAGTTCGTCGTCCAACATTATCATGGCCTCGTGAAATCCCGTTAGTAAAAGGTGGTAAACCTGACGTTGTACAAATTGTTAGGAATTATAATGCTTATCTACGTGCAAGTGATGATTTACCAAAAATGTTTATTGAATCGGACCCAGGATTCTTTTCCAATGCTATTGTTGAAGGTGCCAAGAAGTTTCCTAATACTGAATTTGTCAAAGTAAAAGGTCTTCATTTTTCGCAAGAAGATGCACCTGATGAAATGGGAAAATATATCAAATCGTTCGTTGAGCGAGTTCTCAAAAATGAACAATAATCGCGATTAATTAAGGATCCAGATCTGTGGGCGTGGCTTAAGGGTGGGAAAGAATATATAAGGTGGGGGTCTTATGTAGTTTTGTATCTGTTTTGCAGCAGCCGCCGCCGCCATGAGCACCAACTCGTTTGATGGAAGCATTGTGAGCTCATATTTGACAACGCGCATGCCCCCATGGGCCGGGGTGCGTCAGAATGTGATGGGCTCCAGCATTGATGGTCGCCCCGTCCTGCCCGCAAACTCTACTACCTTGACCTACGAGACCGTGTCTGGAACGCCGTTGGAGACTGCAGCCTCCGCCGCCGCTTCAGCCGCTGCAGCCACCGCCCGCGGGATTGTGACTGACTTTGCTTTCCTGAGCCCGCTTGCAAGCAGTGCAGCTTCCCGTTCATCCGCCCGCGATGACAAGTTGACGGCTCTTTTGGCACAATTGGATTCTTTGACCCGGGAACTTAATGTCGTTTCTCAGCAGCTGTTGGATCTGCGCCAGCAGGTTTCTGCCCTGAAGGCTTCCTCCCCTCCCAATGCGGTTTAAAACATAAATAAAAAACCAGACTCTGTTTGGATTTGGATCAAGCAAGTGTCTTGCTGTCTTTATTTAGGGGTTTTGCGCGCGCGGTAGGCCCGGGACCAGCGGTCTCGGTCGTTGAGGGTCCTGTGTATTTTTTCCAGGACGTGGTAAAGGTGACTCTGGATGTTCAGATACATGGGCATAAGCCCGTCTCTGGGGTGGAGGTAGCACCACTGCAGAGCTTCATGCTGCGGGGTGGTGTTGTAGATGATCCAGTCGTAGCAGGAGCGCTGGGCGTGGTGCCTAAAAATGTCTTTCAGTAGCAAGCTGATTGCCAGGGGCAGGCCCTTGGTGTAAGTGTTTACAAAGCGGTTAAGCTGGGATGGGTGCATACGTGGGGATATGAGATGCATCTTGGACTGTATTTTTAGGTTGGCTATGTTCCCAGCCATATCCCTCCGGGGATTCATGTTGTGCAGAACCACCAGCACAGTGTATCCGGTGCACTTGGGAAATTTGTCATGTAGCTTAGAAGGAAATGCGTGGAAGAACTTGGAGACGCCCTTGTGACCTCCAAGATTTTCCATGCATTCGTCCATAATGATGGCAATGGGCCCACGGGCGGCGGCCTGGGCGAAGATATTTCTGGGATCACTAACGTCATAGTTGTGTTCCAGGATGAGATCGTCATAGGCCATTTTTACAAAGCGCGGGCGGAGGGTGCCAGACTGCGGTATAATGGTTCCATCCGGCCCAGGGGCGTAGTTACCCTCACAGATTTGCATTTCCCACGCTTTGAGTTCAGATGGGGGGATCATGTCTACCTGCGGGGCGATGAAGAAAACGGTTTCCGGGGTAGGGGAGATCAGCTGGGAAGAAAGCAGGTTCCTGAGCAGCTGCGACTTACCGCAGCCGGTGGGCCCGTAAATCACACCTATTACCGGGTGCAACTGGTAGTTAAGAGAGCTGCAGCTGCCGTCATCCCTGAGCAGGGGGGCCACTTCGTTAAGCATGTCCCTGACTCGCATGTTTTCCCTGACCAAATCCGCCAGAAGGCGCTCGCCGCCCAGCGATAGCAGTTCTTGCAAGGAAGCAAAGTTTTTCAACGGTTTGAGACCGTCCGCCGTAGGCATGCTTTTGAGCGTTTGACCAAGCAGTTCCAGGCGGTCCCACAGCTCGGTCACCTGCTCTACGGCATCTCGATCCAGCATATCTCCTCGTTTCGCGGGTTGGGGCGGCTTTCGCTGTACGGCAGTAGTCGGTGCTCGTCCAGACGGGCCAGGGTCATGTCTTTCCACGGGCGCAGGGTCCTCGTCAGCGTAGTCTGGGTCACGGTGAAGGGGTGCGCTCCGGGCTGCGCGCTGGCCAGGGTGCGCTTGAGGCTGGTCCTGCTGGTGCTGAAGCGCTGCCGGTCTTCGCCCTGCGCGTCGGCCAGGTAGCATTTGACCATGGTGTCATAGTCCAGCCCCTCCGCGGCGTGGCCCTTGGCGCGCAGCTTGCCCTTGGAGGAGGCGCCGCACGAGGGGCAGTGCAGACTTTTGAGGGCGTAGAGCTTGGGCGCGAGAAATACCGATTCCGGGGAGTAGGCATCCGCGCCGCAGGCCCCGCAGACGGTCTCGCATTCCACGAGCCAGGTGAGCTCTGGCCGTTCGGGGTCAAAAACCAGGTTTCCCCCATGCTTTTTGATGCGTTTCTTACCTCTGGTTTCCATGAGCCGGTGTCCACGCTCGGTGACGAAAAGGCTGTCCGTGTCCCCGTATACAGACTTGAGAGGCCTGTCCTCGAGCGGTGTTCCGCGGTCCTCCTCGTATAGAAACTCGGACCACTCTGAGACAAAGGCTCGCGTCCAGGCCAGCACGAAGGAGGCTAAGTGGGAGGGGTAGCGGTCGTTGTCCACTAGGGGGTCCACTCGCTCCAGGGTGTGAAGACACATGTCGCCCTCTTCGGCATCAAGGAAGGTGATTGGTTTGTAGGTGTAGGCCACGTGACCGGGTGTTCCTGAAGGGGGGCTATAAAAGGGGGTGGGGGCGCGTTCGTCCTCACTCTCTTCCGCATCGCTGTCTGCGAGGGCCAGCTGTTGGGGTGAGTACTCCCTCTGAAAAGCGGGCATGACTTCTGCGCTAAGATTGTCAGTTTCCAAAAACGAGGAGGATTTGATATTCACCTGGCCCGCGGTGATGCCTTTGAGGGTGGCCGCATCCATCTGGTCAGAAAAGACAATCTTTTTGTTGTCAAGCTTGGTGGCAAACGACCCGTAGAGGGCGTTGGACAGCAACTTGGCGATGGAGCGCAGGGTTTGGTTTTTGTCGCGATCGGCGCGCTCCTTGGCCGCGATGTTTAGCTGCACGTATTCGCGCGCAACGCACCGCCATTCGGGAAAGACGGTGGTGCGCTCGTCGGGCACCAGGTGCACGCGCCAACCGCGGTTGTGCAGGGTGACAAGGTCAACGCTGGTGGCTACCTCTCCGCGTAGGCGCTCGTTGGTCCAGCAGAGGCGGCCGCCCTTGCGCGAGCAGAATGGCGGTAGGGGGTCTAGCTGCGTCTCGTCCGGGGGGTCTGCGTCCACGGTAAAGACCCCGGGCAGCAGGCGCGCGTCGAAGTAGTCTATCTTGCATCCTTGCAAGTCTAGCGCCTGCTGCCATGCGCGGGCGGCAAGCGCGCGCTCGTATGGGTTGAGTGGGGGACCCCATGGCATGGGGTGGGTGAGCGCGGAGGCGTACATGCCGCAAATGTCGTAAACGTAGAGGGGCTCTCTGAGTATTCCAAGATATGTAGGGTAGCATCTTCCACCGCGGATGCTGGCGCGCACGTAATCGTATAGTTCGTGCGAGGGAGCGAGGAGGTCGGGACCGAGGTTGCTACGGGCGGGCTGCTCTGCTCGGAAGACTATCTGCCTGAAGATGGCATGTGAGTTGGATGATATGGTTGGACGCTGGAAGACGTTGAAGCTGGCGTCTGTGAGACCTACCGCGTCACGCACGAAGGAGGCGTAGGAGTCGCGCAGCTTGTTGACCAGCTCGGCGGTGACCTGCACGTCTAGGGCGCAGTAGTCCAGGGTTTCCTTGATGATGTCATACTTATCCTGTCCCTTTTTTTTCCACAGCTCGCGGTTGAGGACAAACTCTTCGCGGTCTTTCCAGTACTCTTGGATCGGAAACCCGTCGGCCTCCGAACGGTAAGAGCCTAGCATGTAGAACTGGTTGACGGCCTGGTAGGCGCAGCATCCCTTTTCTACGGGTAGCGCGTATGCCTGCGCGGCCTTCCGGAGCGAGGTGTGGGTGAGCGCAAAGGTGTCCCTGACCATGACTTTGAGGTACTGGTATTTGAAGTCAGTGTCGTCGCATCCGCCCTGCTCCCAGAGCAAAAAGTCCGTGCGCTTTTTGGAACGCGGATTTGGCAGGGCGAAGGTGACATCGTTGAAGAGTATCTTTCCCGCGCGAGGCATAAAGTTGCGTGTGATGCGGAAGGGTCCCGGCACCTCGGAACGGTTGTTAATTACCTGGGCGGCGAGCACGATCTCGTCAAAGCCGTTGATGTTGTGGCCCACAATGTAAAGTTCCAAGAAGCGCGGGATGCCCTTGATGGAAGGCAATTTTTTAAGTTCCTCGTAGGTGAGCTCTTCAGGGGAGCTGAGCCCGTGCTCTGAAAGGGCCCAGTCTGCAAGATGAGGGTTGGAAGCGACGAATGAGCTCCACAGGTCACGGGCCATTAGCATTTGCAGGTGGTCGCGAAAGGTCCTAAACTGGCGACCTATGGCCATTTTTTCTGGGGTGATGCAGTAGAAGGTAAGCGGGTCTTGTTCCCAGCGGTCCCATCCAAGGTTCGCGGCTAGGTCTCGCGCGGCAGTCACTAGAGGCTCATCTCCGCCGAACTTCATGACCAGCATGAAGGGCACGAGCTGCTTCCCAAAGGCCCCCATCCAAGTATAGGTCTCTACATCGTAGGTGACAAAGAGACGCTCGGTGCGAGGATGCGAGCCGATCGGGAAGAACTGGATCTCCCGCCACCAATTGGAGGAGTGGCTATTGATGTGGTGAAAGTAGAAGTCCCTGCGACGGGCCGAACACTCGTGCTGGCTTTTGTAAAAACGTGCGCAGTACTGGCAGCGGTGCACGGGCTGTACATCCTGCACGAGGTTGACCTGACGACCGCGCACAAGGAAGCAGAGTGGGAATTTGAGCCCCTCGCCTGGCGGGTTTGGCTGGTGGTCTTCTACTTCGGCTGCTTGTCCTTGACCGTCTGGCTGCTCGAGGGGAGTTACGGTGGATCGGACCACCACGCCGCGCGAGCCCAAAGTCCAGATGTCCGCGCGCGGCGGTCGGAGCTTGATGACAACATCGCGCAGATGGGAGCTGTCCATGGTCTGGAGCTCCCGCGGCGTCAGGTCAGGCGGGAGCTCCTGCAGGTTTACCTCGCATAGACGGGTCAGGGCGCGGGCTAGATCCAGGTGATACCTAATTTCCAGGGGCTGGTTGGTGGCGGCGTCGATGGCTTGCAAGAGGCCGCATCCCCGCGGCGCGACTACGGTACCGCGCGGCGGGCGGTGGGCCGCGGGGGTGTCCTTGGATGATGCATCTAAAAGCGGTGACGCGGGCGAGCCCCCGGAGGTAGGGGGGGCTCCGGACCCGCCGGGAGAGGGGGCAGGGGCACGTCGGCGCCGCGCGCGGGCAGGAGCTGGTGCTGCGCGCGTAGGTTGCTGGCGAACGCGACGACGCGGCGGTTGATCTCCTGAATCTGGCGCCTCTGCGTGAAGACGACGGGCCCGGTGAGCTTGAGCCTGAAAGAGAGTTCGACAGAATCAATTTCGGTGTCGTTGACGGCGGCCTGGCGCAAAATCTCCTGCACGTCTCCTGAGTTGTCTTGATAGGCGATCTCGGCCATGAACTGCTCGATCTCTTCCTCCTGGAGATCTCCGCGTCCGGCTCGCTCCACGGTGGCGGCGAGGTCGTTGGAAATGCGGGCCATGAGCTGCGAGAAGGCGTTGAGGCCTCCCTCGTTCCAGACGCGGCTGTAGACCACGCCCCCTTCGGCATCGCGGGCGCGCATGACCACCTGCGCGAGATTGAGCTCCACGTGCCGGGCGAAGACGGCGTAGTTTCGCAGGCGCTGAAAGAGGTAGTTGAGGGTGGTGGCGGTGTGTTCTGCCACGAAGAAGTACATAACCCAGCGTCGCAACGTGGATTCGTTGATATCCCCCAAGGCCTCAAGGCGCTCCATGGCCTCGTAGAAGTCCACGGCGAAGTTGAAAAACTGGGAGTTGCGCGCCGACACGGTTAACTCCTCCTCCAGAAGACGGATGAGCTCGGCGACAGTGTCGCGCACCTCGCGCTCAAAGGCTACAGGGGCCTCTTCTTCTTCTTCAATCTCCTCTTCCATAAGGGCCTCCCCTTCTTCTTCTTCTGGCGGCGGTGGGGGAGGGGGGACACGGCGGCGACGACGGCGCACCGGGAGGCGGTCGACAAAGCGCTCGATCATCTCCCCGCGGCGACGGCGCATGGTCTCGGTGACGGCGCGGCCGTTCTCGCGGGGGCGCAGTTGGAAGACGCCGCCCGTCATGTCCCGGTTATGGGTTGGCGGGGGGCTGCCATGCGGCAGGGATACGGCGCTAACGATGCATCTCAACAATTGTTGTGTAGGTACTCCGCCGCCGAGGGACCTGAGCGAGTCCGCATCGACCGGATCGGAAAACCTCTCGAGAAAGGCGTCTAACCAGTCACAGTCGCAAGGTAGGCTGAGCACCGTGGCGGGCGGCAGCGGGCGGCGGTCGGGGTTGTTTCTGGCGGAGGTGCTGCTGATGATGTAATTAAAGTAGGCGGTCTTGAGACGGCGGATGGTCGACAGAAGCACCATGTCCTTGGGTCCGGCCTGCTGAATGCGCAGGCGGTCGGCCATGCCCCAGGCTTCGTTTTGACATCGGCGCAGGTCTTTGTAGTAGTCTTGCATGAGCCTTTCTACCGGCACTTCTTCTTCTCCTTCCTCTTGTCCTGCATCTCTTGCATCTATCGCTGCGGCGGCGGCGGAGTTTGGCCGTAGGTGGCGCCCTCTTCCTCCCATGCGTGTGACCCCGAAGCCCCTCATCGGCTGAAGCAGGGCTAGGTCGGCGACAACGCGCTCGGCTAATATGGCCTGCTGCACCTGCGTGAGGGTAGACTGGAAGTCATCCATGTCCACAAAGCGGTGGTATGCGCCCGTGTTGATGGTGTAAGTGCAGTTGGCCATAACGGACCAGTTAACGGTCTGGTGACCCGGCTGCGAGAGCTCGGTGTACCTGAGACGCGAGTAAGCCCTCGAGTCAAATACGTAGTCGTTGCAAGTCCGCACCAGGTACTGGTATCCCACCAAAAAGTGCGGCGGCGGCTGGCGGTAGAGGGGCCAGCGTAGGGTGGCCGGGGCTCCGGGGGCGAGATCTTCCAACATAAGGCGATGATATCCGTAGATGTACCTGGACATCCAGGTGATGCCGGCGGCGGTGGTGGAGGCGCGCGGAAAGTCGCGGACGCGGTTCCAGATGTTGCGCAGCGGCAAAAAGTGCTCCATGGTCGGGACGCTCTGGCCGGTCAGGCGCGCGCAATCGTTGACGCTCTAGACCGTGCAAAAGGAGAGCCTGTAAGCGGGCACTCTTCCGTGGTCTGGTGGATAAATTCGCAAGGGTATCATGGCGGACGACCGGGGTTCGAGCCCCGTATCCGGCCGTCCGCCGTGATCCATGCGGTTACCGCCCGCGTGTCGAACCCAGGTGTGCGACGTCAGACAACGGGGGAGTGCTCCTTTTGGCTTCCTTCCAGGCGCGGCGGCTGCTGCGCTAGCTTTTTTGGCCACTGGCCGCGCGCAGCGTAAGCGGTTAGGCTGGAAAGCGAAAGCATTAAGTGGCTCGCTCCCTGTAGCCGGAGGGTTATTTTCCAAGGGTTGAGTCGCGGGACCCCCGGTTCGAGTCTCGGACCGGCCGGACTGCGGCGAACGGGGGTTTGCCTCCCCGTCATGCAAGACCCCGCTTGCAAATTCCTCCGGAAACAGGGACGAGCCCCTTTTTTGCTTTTCCCAGATGCATCCGGTGCTGCGGCAGATGCGCCCCCCTCCTCAGCAGCGGCAAGAGCAAGAGCAGCGGCAGACATGCAGGGCACCCTCCCCTCCTCCTACCGCGTCAGGAGGGGCGACATCCGCGGTTGACGCGGCAGCAGATGGTGATTACGAACCCCCGCGGCGCCGGGCCCGGCACTACCTGGACTTGGAGGAGGGCGAGGGCCTGGCGCGGCTAGGAGCGCCCTCTCCTGAGCGGTACCCAAGGGTGCAGCTGAAGCGTGATACGCGTGAGGCGTACGTGCCGCGGCAGAACCTGTTTCGCGACCGCGAGGGAGAGGAGCCCGAGGAGATGCGGGATCGAAAGTTCCACGCAGGGCGCGAGCTGCGGCATGGCCTGAATCGCGAGCGGTTGCTGCGCGAGGAGGACTTTGAGCCCGACGCGCGAACCGGGATTAGTCCCGCGCGCGCACACGTGGCGGCCGCCGACCTGGTAACCGCATACGAGCAGACGGTGAACCAGGAGATTAACTTTCAAAAAAGCTTTAACAACCACGTGCGTACGCTTGTGGCGCGCGAGGAGGTGGCTATAGGACTGATGCATCTGTGGGACTTTGTAAGCGCGCTGGAGCAAAACCCAAATAGCAAGCCGCTCATGGCGCAGCTGTTCCTTATAGTGCAGCACAGCAGGGACAACGAGGCATTCAGGGATGCGCTGCTAAACATAGTAGAGCCCGAGGGCCGCTGGCTGCTCGATTTGATAAACATCCTGCAGAGCATAGTGGTGCAGGAGCGCAGCTTGAGCCTGGCTGACAAGGTGGCCGCCATCAACTATTCCATGCTTAGCCTGGGCAAGTTTTACGCCCGCAAGATATACCATACCCCTTACGTTCCCATAGACAAGGAGGTAAAGATCGAGGGGTTCTACATGCGCATGGCGCTGAAGGTGCTTACCTTGAGCGACGACCTGGGCGTTTATCGCAACGAGCGCATCCACAAGGCCGTGAGCGTGAGCCGGCGGCGCGAGCTCAGCGACCGCGAGCTGATGCACAGCCTGCAAAGGGCCCTGGCTGGCACGGGCAGCGGCGATAGAGAGGCCGAGTCCTACTTTGACGCGGGCGCTGACCTGCGCTGGGCCCCAAGCCGACGCGCCCTGGAGGCAGCTGGGGCCGGACCTGGGCTGGCGGTGGCACCCGCGCGCGCTGGCAACGTCGGCGGCGTGGAGGAATATGACGAGGACGATGAGTACGAGCCAGAGGACGGCGAGTACTAAGCGGTGATGTTTCTGATCAGATGATGCAAGACGCAACGGACCCGGCGGTGCGGGCGGCGCTGCAGAGCCAGCCGTCCGGCCTTAACTCCACGGACGACTGGCGCCAGGTCATGGACCGCATCATGTCGCTGACTGCGCGCAATCCTGACGCGTTCCGGCAGCAGCCGCAGGCCAACCGGCTCTCCGCAATTCTGGAAGCGGTGGTCCCGGCGCGCGCAAACCCCACGCACGAGAAGGTGCTGGCGATCGTAAACGCGCTGGCCGAAAACAGGGCCATCCGGCCCGACGAGGCCGGCCTGGTCTACGACGCGCTGCTTCAGCGCGTGGCTCGTTACAACAGCGGCAACGTGCAGACCAACCTGGACCGGCTGGTGGGGGATGTGCGCGAGGCCGTGGCGCAGCGTGAGCGCGCGCAGCAGCAGGGCAACCTGGGCTCCATGGTTGCACTAAACGCCTTCCTGAGTACACAGCCCGCCAACGTGCCGCGGGGACAGGAGGACTACACCAACTTTGTGAGCGCACTGCGGCTAATGGTGACTGAGACACCGCAAAGTGAGGTGTACCAGTCTGGGCCAGACTATTTTTTCCAGACCAGTAGACAAGGCCTGCAGACCGTAAACCTGAGCCAGGCTTTCAAAAACTTGCAGGGGCTGTGGGGGGTGCGGGCTCCCACAGGCGACCGCGCGACCGTGTCTAGCTTGCTGACGCCCAACTCGCGCCTGTTGCTGCTGCTAATAGCGCCCTTCACGGACAGTGGCAGCGTGTCCCGGGACACATACCTAGGTCACTTGCTGACACTGTACCGCGAGGCCATAGGTCAGGCGCATGTGGACGAGCATACTTTCCAGGAGATTACAAGTGTCAGCCGCGCGCTGGGGCAGGAGGACACGGGCAGCCTGGAGGCAACCCTAAACTACCTGCTGACCAACCGGCGGCAGAAGATCCCCTCGTTGCACAGTTTAAACAGCGAGGAGGAGCGCATTTTGCGCTACGTGCAGCAGAGCGTGAGCCTTAACCTGATGCGCGACGGGGTAACGCCCAGCGTGGCGCTGGACATGACCGCGCGCAACATGGAACCGGGCATGTATGCCTCAAACCGGCCGTTTATCAACCGCCTAATGGACTACTTGCATCGCGCGGCCGCCGTGAACCCCGAGTATTTCACCAATGCCATCTTGAACCCGCACTGGCTACCGCCCCCTGGTTTCTACACCGGGGGATTCGAGGTGCCCGAGGGTAACGATGGATTCCTCTGGGACGACATAGACGACAGCGTGTTTTCCCCGCAACCGCAGACCCTGCTAGAGTTGCAACAGCGCGAGCAGGCAGAGGCGGCGCTGCGAAAGGAAAGCTTCCGCAGGCCAAGCAGCTTGTCCGATCTAGGCGCTGCGGCCCCGCGGTCAGATGCTAGTAGCCCATTTCCAAGCTTGATAGGGTCTCTTACCAGCACTCGCACCACCCGCCCGCGCCTGCTGGGCGAGGAGGAGTACCTAAACAACTCGCTGCTGCAGCCGCAGCGCGAAAAAAACCTGCCTCCGGCATTTCCCAACAACGGGATAGAGAGCCTAGTGGACAAGATGAGTAGATGGAAGACGTACGCGCAGGAGCACAGGGACGTGCCAGGCCCGCGCCCGCCCACCCGTCGTCAAAGGCACGACCGTCAGCGGGGTCTGGTGTGGGAGGACGATGACTCGGCAGACGACAGCAGCGTCCTGGATTTGGGAGGGAGTGGCAACCCGTTTGCGCACCTTCGCCCCAGGCTGGGGAGAATGTTTTAAAAAAAAAAAAGCATGATGCAAAATAAAAAACTCACCAAGGCCATGGCACCGAGCGTTGGTTTTCTTGTATTCCCCTTAGTATGCGGCGCGCGGCGATGTATGAGGAAGGTCCTCCTCCCTCCTACGAGAGTGTGGTGAGCGCGGCGCCAGTGGCGGCGGCGCTGGGTTCTCCCTTCGATGCTCCCCTGGACCCGCCGTTTGTGCCTCCGCGGTACCTGCGGCCTACCGGGGGGAGAAACAGCATCCGTTACTCTGAGTTGGCACCCCTATTCGACACCACCCGTGTGTACCTGGTGGACAACAAGTCAACGGATGTGGCATCCCTGAACTACCAGAACGACCACAGCAACTTTCTGACCACGGTCATTCAAAACAATGACTACAGCCCGGGGGAGGCAAGCACACAGACCATCAATCTTGACGACCGGTCGCACTGGGGCGGCGACCTGAAAACCATCCTGCATACCAACATGCCAAATGTGAACGAGTTCATGTTTACCAATAAGTTTAAGGCGCGGGTGATGGTGTCGCGCTTGCCTACTAAGGACAATCAGGTGGAGCTGAAATACGAGTGGGTGGAGTTCACGCTGCCCGAGGGCAACTACTCCGAGACCATGACCATAGACCTTATGAACAACGCGATCGTGGAGCACTACTTGAAAGTGGGCAGACAGAACGGGGTTCTGGAAAGCGACATCGGGGTAAAGTTTGACACCCGCAACTTCAGACTGGGGTTTGACCCCGTCACTGGTCTTGTCATGCCTGGGGTATATACAAACGAAGCCTTCCATCCAGACATCATTTTGCTGCCAGGATGCGGGGTGGACTTCACCCACAGCCGCCTGAGCAACTTGTTGGGCATCCGCAAGCGGCAACCCTTCCAGGAGGGCTTTAGGATCACCTACGATGATCTGGAGGGTGGTAACATTCCCGCACTGTTGGATGTGGACGCCTACCAGGCGAGCTTGAAAGATGACACCGAACAGGGCGGGGGTGGCGCAGGCGGCAGCAACAGCAGTGGCAGCGGCGCGGAAGAGAACTCCAACGCGGCAGCCGCGGCAATGCAGCCGGTGGAGGACATGAACGATCATGCCATTCGCGGCGACACCTTTGCCACACGGGCTGAGGAGAAGCGCGCTGAGGCCGAAGCAGCGGCCGAAGCTGCCGCCCCCGCTGCGCAACCCGAGGTCGAGAAGCCTCAGAAGAAACCGGTGATCAAACCCCTGACAGAGGACAGCAAGAAACGCAGTTACAACCTAATAAGCAATGACAGCACCTTCACCCAGTACCGCAGCTGGTACCTTGCATACAACTACGGCGACCCTCAGACCGGAATCCGCTCATGGACCCTGCTTTGCACTCCTGACGTAACCTGCGGCTCGGAGCAGGTCTACTGGTCGTTGCCAGACATGATGCAAGACCCCGTGACCTTCCGCTCCACGCGCCAGATCAGCAACTTTCCGGTGGTGGGCGCCGAGCTGTTGCCCGTGCACTCCAAGAGCTTCTACAACGACCAGGCCGTCTACTCCCAACTCATCCGCCAGTTTACCTCTCTGACCCACGTGTTCAATCGCTTTCCCGAGAACCAGATTTTGGCGCGCCCGCCAGCCCCCACCATCACCACCGTCAGTGAAAACGTTCCTGCTCTCACAGATCACGGGACGCTACCGCTGCGCAACAGCATCGGAGGAGTCCAGCGAGTGACCATTACTGACGCCAGACGCCGCACCTGCCCCTACGTTTACAAGGCCCTGGGCATAGTCTCGCCGCGCGTCCTATCGAGCCGCACTTTTTGAGCAAGCATGTCCATCCTTATATCGCCCAGCAATAACACAGGCTGGGGCCTGCGCTTCCCAAGCAAGATGTTTGGCGGGGCCAAGAAGCGCTCCGACCAACACCCAGTGCGCGTGCGCGGGCACTACCGCGCGCCCTGGGGCGCGCACAAACGCGGCCGCACTGGGCGCACCACCGTCGATGACGCCATCGACGCGGTGGTGGAGGAGGCGCGCAACTACACGCCCACGCCGCCACCAGTGTCCACAGTGGACGCGGCCATTCAGACCGTGGTGCGCGGAGCCCGGCGCTATGCTAAAATGAAGAGACGGCGGAGGCGCGTAGCACGTCGCCACCGCCGCCGACCCGGCACTGCCGCCCAACGCGCGGCGGCGGCCCTGCTTAACCGCGCACGTCGCACCGGCCGACGGGCGGCCATGCGGGCCGCTCGAAGGCTGGCCGCGGGTATTGTCACTGTGCCCCCCAGGTCCAGGCGACGAGCGGCCGCCGCAGCAGCCGCGGCCATTAGTGCTATGACTCAGGGTCGCAGGGGCAACGTGTATTGGGTGCGCGACTCGGTTAGCGGCCTGCGCGTGCCCGTGCGCACCCGCCCCCCGCGCAACTAGATTGCAAGAAAAAACTACTTAGACTCGTACTGTTGTATGTATCCAGCGGCGGCGGCGCGCAACGAAGCTATGTCCAAGCGCAAAATCAAAGAAGAGATGCTCCAGGTCATCGCGCCGGAGATCTATGGCCCCCCGAAGAAGGAAGAGCAGGATTACAAGCCCCGAAAGCTAAAGCGGGTCAAAAAGAAAAAGAAAGATGATGATGATGAACTTGACGACGAGGTGGAACTGCTGCACGCTACCGCGCCCAGGCGACGGGTACAGTGGAAAGGTCGACGCGTAAAACGTGTTTTGCGACCCGGCACCACCGTAGTCTTTACGCCCGGTGAGCGCTCCACCCGCACCTACAAGCGCGTGTATGATGAGGTGTACGGCGACGAGGACCTGCTTGAGCAGGCCAACGAGCGCCTCGGGGAGTTTGCCTACGGAAAGCGGCATAAGGACATGCTGGCGTTGCCGCTGGACGAGGGCAACCCAACACCTAGCCTAAAGCCCGTAACACTGCAGCAGGTGCTGCCCGCGCTTGCACCGTCCGAAGAAAAGCGCGGCCTAAAGCGCGAGTCTGGTGACTTGGCACCCACCGTGCAGCTGATGGTACCCAAGCGCCAGCGACTGGAAGATGTCTTGGAAAAAATGACCGTGGAACCTGGGCTGGAGCCCGAGGTCCGCGTGCGGCCAATCAAGCAGGTGGCGCCGGGACTGGGCGTGCAGACCGTGGACGTTCAGATACCCACTACCAGTAGCACCAGTATTGCCACCGCCACAGAGGGCATGGAGACACAAACGTCCCCGGTTGCCTCAGCGGTGGCGGATGCCGCGGTGCAGGCGGTCGCTGCGGCCGCGTCCAAGACCTCTACGGAGGTGCAAACGGACCCGTGGATGTTTCGCGTTTCAGCCCCCCGGCGCCCGCGCGGTTCGAGGAAGTACGGCGCCGCCAGCGCGCTACTGCCCGAATATGCCCTACATCCTTCCATTGCGCCTACCCCCGGCTATCGTGGCTACACCTACCGCCCCAGAAGACGAGCAACTACCCGACGCCGAACCACCACTGGAACCCGCCGCCGCCGTCGCCGTCGCCAGCCCGTGCTGGCCCCGATTTCCGTGCGCAGGGTGGCTCGCGAAGGAGGCAGGACCCTGGTGCTGCCAACAGCGCGCTACCACCCCAGCATCGTTTAAAAGCCGGTCTTTGTGGTTCTTGCAGATATGGCCCTCACCTGCCGCCTCCGTTTCCCGGTGCCGGGATTCCGAGGAAGAATGCACCGTAGGAGGGGCATGGCCGGCCACGGCCTGACGGGCGGCATGCGTCGTGCGCACCACCGGCGGCGGCGCGCGTCGCACCGTCGCATGCGCGGCGGTATCCTGCCCCTCCTTATTCCACTGATCGCCGCGGCGATTGGCGCCGTGCCCGGAATTGCATCCGTGGCCTTGCAGGCGCAGAGACACTGATTAAAAACAAGTTGCATGTGGAAAAATCAAAATAAAAAGTCTGGACTCTCACGCTCGCTTGGTCCTGTAACTATTTTGTAGAATGGAAGACATCAACTTTGCGTCTCTGGCCCCGCGACACGGCTCGCGCCCGTTCATGGGAAACTGGCAAGATATCGGCACCAGCAATATGAGCGGTGGCGCCTTCAGCTGGGGCTCGCTGTGGAGCGGCATTAAAAATTTCGGTTCCACCGTTAAGAACTATGGCAGCAAGGCCTGGAACAGCAGCACAGGCCAGATGCTGAGGGATAAGTTGAAAGAGCAAAATTTCCAACAAAAGGTGGTAGATGGCCTGGCCTCTGGCATTAGCGGGGTGGTGGACCTGGCCAACCAGGCAGTGCAAAATAAGATTAACAGTAAGCTTGATCCCCGCCCTCCCGTAGAGGAGCCTCCACCGGCCGTGGAGACAGTGTCTCCAGAGGGGCGTGGCGAAAAGCGTCCGCGCCCCGACAGGGAAGAAACTCTGGTGACGCAAATAGACGAGCCTCCCTCGTACGAGGAGGCACTAAAGCAAGGCCTGCCCACCACCCGTCCCATCGCGCCCATGGCTACCGGAGTGCTGGGCCAGCACACACCCGTAACGCTGGACCTGCCTCCCCCCGCCGACACCCAGCAGAAACCTGTGCTGCCAGGCCCGACCGCCGTTGTTGTAACCCGTCCTAGCCGCGCGTCCCTGCGCCGCGCCGCCAGCGGTCCGCGATCGTTGCGGCCCGTAGCCAGTGGCAACTGGCAAAGCACACTGAACAGCATCGTGGGTCTGGGGGTGCAATCCCTGAAGCGCCGACGATGCTTCTGAATAGCTAACGTGTCGTATGTGTGTCATGTATGCGTCCATGTCGCCGCCAGAGGAGCTGCTGAGCCGCCGCGCGCCCGCTTTCCAAGATGGCTACCCCTTCGATGATGCCGCAGTGGTCTTACATGCACATCTCGGGCCAGGACGCCTCGGAGTACCTGAGCCCCGGGCTGGTGCAGTTTGCCCGCGCCACCGAGACGTACTTCAGCCTGAATAACAAGTTTAGAAACCCCACGGTGGCGCCTACGCACGACGTGACCACAGACCGGTCCCAGCGTTTGACGCTGCGGTTCATCCCTGTGGACCGTGAGGATACTGCGTACTCGTACAAGGCGCGGTTCACCCTAGCTGTGGGTGATAACCGTGTGCTGGACATGGCTTCCACGTACTTTGACATCCGCGGCGTGCTGGACAGGGGCCCTACTTTTAAGCCCTACTCTGGCACTGCCTACAACGCCCTGGCTCCCAAGGGTGCCCCAAATCCTTGCGAATGGGATGAAGCTGCTACTGCTCTTGAAATAAACCTAGAAGAAGAGGACGATGACAACGAAGACGAAGTAGACGAGCAAGCTGAGCAGCAAAAAACTCACGTATTTGGGCAGGCGCCTTATTCTGGTATAAATATTACAAAGGAGGGTATTCAAATAGGTGTCGAAGGTCAAACACCTAAATATGCCGATAAAACATTTCAACCTGAACCTCAAATAGGAGAATCTCAGTGGTACGAAACTGAAATTAATCATGCAGCTGGGAGAGTCCTTAAAAAGACTACCCCAATGAAACCATGTTACGGTTCATatgcaaaacccacaaatgaaaatggagggcaaggcattcttgtaaagcaacaaaatggaaagctagaaagtcaagtggaaatgcaatttttctcaactactgaggcgaccgcaggcaatggtgataacttgactcctaaagtggtattgtacagtgaagatgtagatatagaaaccccagacactcatatttcttacatgcccactattaaggaaggtaactcacgagaactaatgggccaacaatctatgcccaacaggcctaattacattgcttttagggacaattttattggtctaatgtattacaacagcacgggtaatatgggtgttctggcgggccaagcatcgcagttgaatgctgttgtagatttgcaagacagaaacacagagctttcataccagcttttgcttgattccattggtgatagaaccaggtacttttctatgtggaatcaggctgttgacagctatgatccagatgttagaattattgaaaatcatggaactgaagatgaacttccaaattactgctttccactgggaggtgtgattaatacagagactcttaccaaggtaaaacctaaaacaggtcaggaaaatggatgggaaaaagatgctacagaattttcagataaaaatgaaataagagttggaaataattttgccatggaaatcaatctaaatgccaacctgtggagaaatttcctgtactccaacatagcgctgtatttgcccgacaagctaaagtacagtccttccaacgtaaaaatttctgataacccaaacacctacgactacatgaacaagcgagtggtggctcccgggttagtggactgctacattaaccttggagcacgctggtcccttgactatatggacaacgtcaacccatttaaccaccaccgcaatgctggcctgcgctaccgctcaatgttgctgggcaatggtcgctatgtgcccttccacatccaggtgcctcagaagttctttgccattaaaaacctccttctcctgccgggctcatacacctacgagtggaacttcaggaaggatgttaacatggttctgcagagctccctaggaaatgacctaagggttgacggagccagcattaagtttgatagcatttgcctttacgccaccttcttccccatggcccacaacaccgcctccacgcttgaggccatgcttagaaacgacaccaacgaccagtcctttaacgactatctctccgccgccaacatgctctaccctatacccgccaacgctaccaacgtgcccatatccatcccctcccgcaactgggcggctttccgcggctgggccttcacgcgccttaagactaaggaaaccccatcactgggctcgggctacgacccttattacacctactctggctctataccctacctagatggaaccttttacctcaaccacacctttaagaaggtggccattacctttgactcttctgtcagctggcctggcaatgaccgcctgcttacccccaacgagtttgaaattaagcgctcagttgacggggagggttacaacgttgcccagtgtaacatgaccaaagactggttcctggtacaaatgctagctaactacaacattggctaccagggcttctatatcccagagagctacaaggaccgcatgtactccttctttagaaacttccagcccatgagccgtcaggtggtggatgatactaaatacaaggactaccaacaggtgggcatcctacaccaacacaacaactctggatttgttggctaccttgcccccaccatgcgcgaaggacaggcctaccctgctaacttcccctatccgcttataggcaagaccgcagttgacagcattacccagaaaaagtttctttgcgatcgcaccctttggcgcatcccattctccagtaactttatgtccatgggcgcactcacagacctgggccaaaaccttctctacgccaactccgcccacgcgctagacatgacttttgaggtggatcccatggacgagcccacccttctttatgttttgtttgaagtctttgacgtggtccgtgtgcaccggccgcaccgcggcgtcatcgaaaccgtgtacctgcgcacgcccttctcggccggcaacgccacaacataaagaagcaagcaacatcaacaacagctgccgccatgggctccagtgagcaggaactgaaagccattgtcaaagatcttggttgtgggccatattttttgggcacctatgacaagcgctttccaggctttgtttctccacacaagctcgcctgcgccatagtcaatacggccggtcgcgagactgggggcgtacactggatggcctttgcctggaacccgcactcaaaaacatgctacctctttgagccctttggcttttctgaccagcgactcaagcaggtttaccagtttgagtacgagtcactcctgcgccgtagcgccattgcttcttcccccgaccgctgtataacgctggaaaagtccacccaaagcgtacaggggcccaactcggccgcctgtggactattctgctgcatgtttctccacgcctttgccaactggccccaaactcccatggatcacaaccccaccatgaaccttattaccggggtacccaactccatgctcaacagtccccaggtacagcccaccctgcgtcgcaaccaggaacagctctacagcttcctggagcgccactcgccctacttccgcagccacagtgcgcagattaggagcgccacttctttttgtcacttgaaaaacatgtaaaaataatgtactagagacactttcaataaaggcaaatgcttttatttgtacactctcgggtgattatttacccccacccttgccgtctgcgccgtttaaaaatcaaaggggttctgccgcgcatcgctatgcgccactggcagggacacgttgcgatactggtgtttagtgctccacttaaactcaggcacaaccatccgcggcagctcggtgaagttttcactccacaggctgcgcaccatcaccaacgcgtttagcaggtcgggcgccgatatcttgaagtcgcagttggggcctccgccctgcgcgcgcgagttgcgatacacagggttgcagcactggaacactatcagcgccgggtggtgcacgctggccagcacgctcttgtcggagatcagatccgcgtccaggtcctccgcgttgctcagggcgaacggagtcaactttggtagctgccttcccaaaaagggcgcgtgcccaggctttgagttgcactcgcaccgtagtggcatcaaaaggtgaccgtgcccggtctgggcgttaggatacagcgcctgcataaaagccttgatctgcttaaaagccacctgagcctttgcgccttcagagaagaacatgccgcaagacttgccggaaaactgattggccggacaggccgcgtcgtgcacgcagcaccttgcgtcggtgttggagatctgcaccacatttcggccccaccggttcttcacgatcttggccttgctagactgctccttcagcgcgcgctgcccgttttcgctcgtcacatccatttcaatcacgtgctccttatttatcataatgcttccgtgtagacacttaagctcgccttcgatctcagcgcagcggtgcagccacaacgcgcagcccgtgggctcgtgatgcttgtaggtcacctctgcaaacgactgcaggtacgcctgcaggaatcgccccatcatcgtcacaaaggtcttgttgctggtgaaggtcagctgcaacccgcggtgctcctcgttcagccaggtcttgcatacggccgccagagcttccacttggtcaggcagtagtttgaagttcgcctttagatcgttatccacgtggtacttgtccatcagcgcgcgcgcagcctccatgcccttctcccacgcagacacgatcggcacactcagcgggttcatcaccgtaatttcactttccgcttcgctgggctcttcctcttcctcttgcgtccgcataccacgcgccactgggtcgtcttcattcagccgccgcactgtgcgcttacctcctttgccatgcttgattagcaccggtgggttgctgaaacccaccatttgtagcgccacatcttctctttcttcctcgctgtccacgattacctctggtgatggcgggcgctcgggcttgggagaagggcgcttctttttcttcttgggcgcaatggccaaatccgccgccgaggtcgatggccgcgggctgggtgtgcgcggcaccagcgcgtcttgtgatgagtcttcctcgtcctcggactcgatacgccgcctcatccgcttttttgggggcgcccggggaggcggcggcgacggggacggggacgacacgtcctccatggttgggggacgtcgcgccgcaccgcgtccgcgctcgggggtggtttcgcgctgctcctcttcccgactggccatttccttctcctataggcagaaaaagatcatggagtcagtcgagaagaaggacagcctaaccgccccctctgagttcgccaccaccgcctccaccgatgccgccaacgcgcctaccaccttccccgtcgaggcacccccgcttgaggaggaggaagtgattatcgagcaggacccaggttttgtaagcgaagacgacgaggaccgctcagtaccaacagaggataaaaagcaagaccaggacaacgcagaggcaaacgaggaacaagtcgggcggggggacgaaaggcatggcgactacctagatgtgggagacgacgtgctgttgaagcatctgcagcgccagtgcgccattatctgcgacgcgttgcaagagcgcagcgatgtgcccctcgccatagcggatgtcagccttgcctacgaacgccacctattctcaccgcgcgtaccccccaaacgccaagaaaacggcacatgcgagcccaacccgcgcctcaacttctaccccgtatttgccgtgccagaggtgcttgccacctatcacatctttttccaaaactgcaagatacccctatcctgccgtgccaaccgcagccgagcggacaagcagctggccttgcggcagggcgctgtcatacctgatatcgcctcgctcaacgaagtgccaaaaatctttgagggtcttggacgcgacgagaagcgcgcggcaaacgctctgcaacaggaaaacagcgaaaatgaaagtcactctggagtgttggtggaactcgagggtgacaacgcgcgcctagccgtactaaaacgcagcatcgaggtcacccactttgcctacccggcacttaacctaccccccaaggtcatgagcacagtcatgagtgagctgatcgtgcgccgtgcgcagcccctggagagggatgcaaatttgcaagaacaaacagaggagggcctacccgcagttggcgacgagcagctagcgcgctggcttcaaacgcgcgagcctgccgacttggaggagcgacgcaaactaatgatggccgcagtgctcgttaccgtggagcttgagtgcatgcagcggttctttgctgacccggagatgcagcgcaagctagaggaaacattgcactacacctttcgacagggctacgtacgccaggcctgcaagatctccaacgtggagctctgcaacctggtctcctaccttggaattttgcacgaaaaccgccttgggcaaaacgtgcttcattccacgctcaagggcgaggcgcgccgcgactacgtccgcgactgcgtttacttatttctatgctacacctggcagacggccatgggcgtttggcagcagtgcttggaggagtgcaacctcaaggagctgcagaaactgctaaagcaaaacttgaaggacctatggacggccttcaacgagcgctccgtggccgcgcacctggcggacatcattttccccgaacgcctgcttaaaaccctgcaacagggtctgccagacttcaccagtcaaagcatgttgcagaactttaggaactttatcctagagcgctcaggaatcttgcccgccacctgctgtgcacttcctagcgactttgtgcccattaagtaccgcgaatgccctccgccgctttggggccactgctaccttctgcagctagccaactaccttgcctaccactctgacataatggaagacgtgagcggtgacggtctactggagtgtcactgtcgctgcaacctatgcaccccgcaccgctccctggtttgcaattcgcagctgcttaacgaaagtcaaattatcggtacctttgagctgcagggtccctcgcctgacgaaaagtccgcggctccggggttgaaactcactccggggctgtggacgtcggcttaccttcgcaaatttgtacctgaggactaccacgcccacgagattaggttctacgaagaccaatcccgcccgccaaatgcggagcttaccgcctgcgtcattacccagggccacattcttggccaattgcaagccatcaacaaagcccgccaagagtttctgctacgaaagggacggggggtttacttggacccccagtccggcgaggagctcaacccaatccccccgccgccgcagccctatcagcagcagccgcgggcccttgcttcccaggatggcacccaaaaagaagctgcagctgccgccgccacccacggacgaggaggaatactgggacagtcaggcagaggaggttttggacgaggaggaggaggacatgatggaagactgggagagcctagacgaggaagcttccgaggtcgaagaggtgtcagacgaaacaccgtcaccctcggtcgcattcccctcgccggcgccccagaaatcggcaaccggttccagcatggctacaacctccgctcctcaggcgccgccggcactgcccgttcgccgacccaaccgtagatgggacaccactggaaccagggccggtaagtccaagcagccgccgccgttagcccaagagcaacaacagcgccaaggctaccgctcatggcgcgggcacaagaacgccatagttgcttgcttgcaagactgtgggggcaacatctccttcgcccgccgctttcttctctaccatcacggcgtggccttcccccgtaacatcctgcattactaccgtcatctctacagcccatactgcaccggcggcagcggcagcggcagcaacagcagcggccacacagaagcaaaggcgaccggatagcaagactctgacaaagcccaagaaatccacagcggcggcagcagcaggaggaggagcgctgcgtctggcgcccaacgaacccgtatcgacccgcgagcttagaaacaggatttttcccactctgtatgctatatttcaacagagcaggggccaagaacaagagctgaaaataaaaaacaggtctctgcgatccctcacccgcagctgcctgtatcacaaaagcgaagatcagcttcggcgcacgctggaagacgcggaggctctcttcagtaaatactgcgcgctgactcttaaggactagtttcgcgccctttctcaaatttaagcgcgaaaactacgtcatctccagcggccacacccggcgccagcacctgtcgtcagcgccattatgagcaaggaaattcccacgccctacatgtggagttaccagccacaaatgggacttgcggctggagctgcccaagactactcaacccgaataaactacatgagcgcgggaccccacatgatatcccgggtcaacggaatccgcgcccaccgaaaccgaattctcttggaacaggcggctattaccaccacacctcgtaataaccttaatccccgtagttggcccgctgccctggtgtaccaggaaagtcccgctcccaccactgtggtacttcccagagacgcccaggccgaagttcagatgactaactcaggggcgcagcttgcgggcggctttcgtcacagggtgcggtcgcccgggcagggtataactcacctgacaatcagagggcgaggtattcagctcaacgacgagtcggtgagctcctcgcttggtctccgtccggacgggacatttcagatcggcggcgccggccgtccttcattcacgcctcgtcaggcaatcctaactctgcagacctcgtcctctgagccgcgctctggaggcattggaactctgcaatttattgaggagtttgtgccatcggtctactttaaccccttctcgggacctcccggccactatccggatcaatttattcctaactttgacgcggtaaaggactcggcggacggctacgactgaatgttaagtggagaggcagagcaactgcgcctgaaacacctggtccactgtcgccgccacaagtgctttgcccgcgactccggtgagttttgctactttgaattgcccgaggatcatatcgagggcccggcgcacggcgtccggcttaccgcccagggagagcttgcccgtagcctgattcgggagtttacccagcgccccctgctagttgagcgggacaggggaccctgtgttctcactgtgatttgcaactgtcctaaccttggattacatcaagatcctctagttaatattaactagagtacccggggatcttattccctttaactaataaaaaaaaataataaagcatcacttacttaaaatcagttagcaaatttctgtccagtttattcagcagcacctccttgccctcctcccagctctggtattgcagcttcctcctggctgcaaactttctccacaatctaaatggaatgtcagtttcctcctgttcctgtccatccgcacccactatcttcatgttgttgcagatgaagcgcgcaagaccgtctgaagataccttcaaccccgtgtatccatATGACACGGAAACCGGTCCTCCAACTGTGCCTTTTCTTACTCCTCCCTTTGTATCCCCCAATGGGTTTCAAGAGAGTCCCCCTGGGGTACTCTCTTTGCGCCTATCCGAACCTCTAGTTACCTCCAATGGCATGCTTGCGCTCAAAATGGGCAACGGCCTCTCTCTGGACGAGGCCGGCAACCTTACCTCCCAAAATGTAACCACTGTGAGCCCACCTCTCAAAAAAACCAAGTCAAACATAAACCTGGAAATATCTGCACCCCTCACAGTTACCTCAGAAGCCCTAACTGTGGCTGCCGCCGCACCTCTAATGGTCGCGGGCAACACACTCACCATGCAATCACAGGCCCCGCTAACCGTGCACGACTCCAAACTTAGCATTGCCACCCAAGGACCCCTCACAGTGTCAGAAGGAAAGCTAGCCCTGCAAACATCAGGCCCCCTCACCACCACCGATAGCAGTACCCTTACTATCACTGCCTCACCCCCTCTAACTACTGCCACTGGTAGCTTGGGCATTGACTTGAAAGAGCCCATTTATACACAAAATGGAAAACTAGGACTAAAGTACGGGGCTCCTTTGCATGTAACAGACGACCTAAACACTTTGACCGTAGCAACTGGTCCAGGTGTGACTATTAATAATACTTCCTTGCAAACTAAAGTTACTGGAGCCTTGGGTTTTGATTCACAAGGCAATATGCAACTTAATGTAGCAGGAGGACTAAGGATTGATTCTCAAAACAGACGCCTTATACTTGATGTTAGTTATCCGTTTGATGCTCAAAACCAACTAAATCTAAGACTAGGACAGGGCCCTCTTTTTATAAACTCAGCCCACAACTTGGATATTAACTACAACAAAGGCCTTTACTTGTTTACAGCTTCAAACAATTCCAAAAAGCTTGAGGTTAACCTAAGCACTGCCAAGGGGTTGATGTTTGACGCTACAGCCATAGCCATTAATGCAGGAGATGGGCTTGAATTTGGTTCACCTAATGCACCAAACACAAATCCCCTCAAAACAAAAATTGGCCATGGCCTAGAATTTGATTCAAACAAGGCTATGGTTCCTAAACTAGGAACTGGCCTTAGTTTTGACAGCACAGGTGCCATTACAGTAGGAAACAAAAATAATGATAAGCTAACTTTGTGGACCACACCAGCTCCATCTCCTAACTGTAGACTAAATGCAGAGAAAGATGCTAAACTCACTTTGGTCTTAACAAAATGTGGCAGTCAAATACTTGCTACAGTTTCAGTTTTGGCTGTTAAAGGCAGTTTGGCTCCAATATCTGGAACAGTTCAAAGTGCTCATCTTATTATAAGATTTGACGAAAATGGAGTGCTACTAAACAATTCCTTCCTGGACCCAGAATATTGGAACTTTAGAAATGGAGATCTTACTGAAGGCACAGCCTATACAAACGCTGTTGGATTTATGCCTAACCTATCAGCTTATCCAAAATCTCACGGTAAAACTGCCAAAAGTAACATTGTCAGTCAAGTTTACTTAAACGGAGACAAAACTAAACCTGTAACACTAACCATTACACTAAACGGTACACAGGAAACAGGAGACACAACTCCAAGTGCATACTCTATGTCATTTTCATGGGACTGGTCTGGCCACAACTACATTAATGAAATATTTGCCACATCCTCTTACACTTTTTCATACATTGCCCAAGAATAAAGAATCGTTTGTGTTATGTTTCAACGTGTTTATTTTTCAATTGCAGAAAATTTCAAGTCATTTTTCATTCAGTAGTATAGCCCCACCACCACATAGCTTATACAGATCACCGTACCTTAATCAAACTCACAGAACCCTAGTATTCAACCTGCCACCTCCCTCCCAACACACAGAGTACACAGTCCTTTCTCCCCGGCTGGCCTTAAAAAGCATCATATCATGGGTAACAGACATATTCTTAGGTGTTATATTCCACACGGTTTCCTGTCGAGCCAAACGCTCATCAGTGATATTAATAAACTCCCCGGGCAGCTCACTTAAGTTCATGTCGCTGTCCAGCTGCTGAGCCACAGGCTGCTGTCCAACTTGCGGTTGCTTAACGGGCGGCGAAGGAGAAGTCCACGCCTACATGGGGGTAGAGTCATAATCGTGCATCAGGATAGGGCGGTGGTGCTGCAGCAGCGCGCGAATAAACTGCTGCCGCCGCCGCTCCGTCCTGCAGGAATACAACATGGCAGTGGTCTCCTCAGCGATGATTCGCACCGCCCGCAGCATAAGGCGCCTTGTCCTCCGGGCACAGCAGCGCACCCTGATCTCACTTAAATCAGCACAGTAACTGCAGCACAGCACCACAATATTGTTCAAAATCCCACAGTGCAAGGCGCTGTATCCAAAGCTCATGGCGGGGACCACAGAACCCACGTGGCCATCATACCACAAGCGCAGGTAGATTAAGTGGCGACCCCTCATAAACACGCTGGACATAAACATTACCTCTTTTGGCATGTTGTAATTCACCACCTCCCGGTACCATATAAACCTCTGATTAAACATGGCGCCATCCACCACCATCCTAAACCAGCTGGCCAAAACCTGCCCGCCGGCTATACACTGCAGGGAACCGGGACTGGAACAATGACAGTGGAGAGCCCAGGACTCGTAACCATGGATCATCATGCTCGTCATGATATCAATGTTGGCACAACACAGGCACACGTGCATACACTTCCTCAGGATTACAAGCTCCTCCCGCGTTAGAACCATATCCCAGGGAACAACCCATTCCTGAATCAGCGTAAATCCCACACTGCAGGGAAGACCTCGCACGTAACTCACGTTGTGCATTGTCAAAGTGTTACATTCGGGCAGCAGCGGATGATCCTCCAGTATGGTAGCGCGGGTTTCTGTCTCAAAAGGAGGTAGACGATCCCTACTGTACGGAGTGCGCCGAGACAACCGAGATCGTGTTGGTCGTAGTGTCATGCCAAATGGAACGCCGGACGTAGTCATATTTCCTGAAGCAAAACCAGGTGCGGGCGTGACAAACAGATCTGCGTCTCCGGTCTCGCCGCTTAGATCGCTCTGTGTAGTAGTTGTAGTATATCCACTCTCTCAAAGCATCCAGGCGCCCCCTGGCTTCGGGTTCTATGTAAACTCCTTCATGCGCCGCTGCCCTGATAACATCCACCACCGCAGAATAAGCCACACCCAGCCAACCTACACATTCGTTCTGCGAGTCACACACGGGAGGAGCGGGAAGAGCTGGAAGAACCATGTTTTTTTTTTTATTCCAAAAGATTATCCAAAACCTCAAAATGAAGATCTATTAAGTGAACGCGCTCCCCTCCGGTGGCGTGGTCAAACTCTACAGCCAAAGAACAGATAATGGCATTTGTAAGATGTTGCACAATGGCTTCCAAAAGGCAAACGGCCCTCACGTCCAAGTGGACGTAAAGGCTAAACCCTTCAGGGTGAATCTCCTCTATAAACATTCCAGCACCTTCAACCATGCCCAAATAATTCTCATCTCGCCACCTTCTCAATATATCTCTAAGCAAATCCCGAATATTAAGTCCGGCCATTGTAAAAATCTGCTCCAGAGCGCCCTCCACCTTCAGCCTCAAGCAGCGAATCATGATTGCAAAAATTCAGGTTCCTCACAGACCTGTATAAGATTCAAAAGCGGAACATTAACAAAAATACCGCGATCCCGTAGGTCCCTTCGCAGGGCCAGCTGAACATAATCGTGCAGGTCTGCACGGACCAGCGCGGCCACTTCCCCGCCAGGAACCTTGACAAAAGAACCCACACTGATTATGACACGCATACTCGGAGCTATGCTAACCAGCGTAGCCCCGATGTAAGCTTTGTTGCATGGGCGGCGATATAAAATGCAAGGTGCTGCTCAAAAAATCAGGCAAAGCCTCGCGCAAAAAAGAAAGCACATCGTAGTCATGCTCATGCAGATAAAGGCAGGTAAGCTCCGGAACCACCACAGAAAAAGACACCATTTTTCTCTCAAACATGTCTGCGGGTTTCTGCATAAACACAAAATAAAATAACAAAAAAACATTTAAACATTAGAAGCCTGTCTTACAACAGGAAAAACAACCCTTATAAGCATAAGACGGACTACGGCCATGCCGGCGTGACCGTAAAAAAACTGGTCACCGTGATTAAAAAGCACCACCGACAGCTCCTCGGTCATGTCCGGAGTCATAATGTAAGACTCGGTAAACACATCAGGTTGATTCATCGGTCAGTGCTAAAAAGCGACCGAAATAGCCCGGGGGAATACATACCCGCAGGCGTAGAGACAACATTACAGCCCCCATAGGAGGTATAACAAAATTAATAGGAGAGAAAAACACATAAACACCTGAAAAACCCTCCTGCCTAGGCAAAATAGCACCCTCCCGCTCCAGAACAACATACAGCGCTTCACAGCGGCAGCCTAACAGTCAGCCTTACCAGTAAAAAAGAAAACCTATTAAAAAAACACCACTCGACACGGCACCAGCTCAATCAGTCACAGTGTAAAAAAGGGCCAAGTGCAGAGCGAGTATATATAGGACTAAAAAATGACGTAACGGTTAAAGTCCACAAAAAACACCCAGAAAACCGCACGCGAACCTACGCCCAGAAACGAAAGCCAAAAAACCCACAACTTCCTCAAATCGTCACTTCCGTTTTCCCACGTTACGTAACTTCCCATTTTAAGAAAACTACAATTCCCAACACATACAAGTTACTCCGCCCTAAAACCTACGTCACCCGCCCCGTTCCCACGCCCCGCGCCACGTCACAAACTCCACCCCCTCATTATCATATTGGCTTCAATCCAAAATAAGGTATATTATTGATGATGATTTAAATGGATCCATTTAAATCGGTACCCAGCTTTTGTTCCCTTTAGTGAGGGTTAATTGCGCGCTTGGCGTAATCATGGTCATAGCTGTTTCCTGTGTGAAATTGTTATCCGCTCACAATTCCACACAACATACGAGCCGGAAGCATAAAGTGTAAAGCCTGGGGTGCCTAATGAGTGAGCTAACTCACATTAATTGCGTTGCGCTCACTGCCCGCTTTCCAGTCGGGAAACCTGTCGTGCCAGCTGCATTAATGAATCGGCCAACGCGCGGGGAGAGGCGGTTTGCGTATTGGGCGCTCTTCCGCTTCCTCGCTCACTGACTCGCTGCGCTCGGTCGTTCGGCTGCGGCGAGCGGTATCAGCTCACTCAAAGGCGGTAATACGGTTATCCACAGAATCAGGGGATAACGCAGGAAAGAACATGTGAGCAAAAGGCCAGCAAAAGGCCAGGAACCGTAAAAAGGCCGCGTTGCTGGCGTTTTTCCATAGGCTCCGCCCCCCTGACGAGCATCACAAAAATCGACGCTCAAGTCAGAGGTGGCGAAACCCGACAGGACTATAAAGATACCAGGCGTTTCCCCCTGGAAGCTCCCTCGTGCGCTCTCCTGTTCCGACCCTGCCGCTTACCGGATACCTGTCCGCCTTTCTCCCTTCGGGAAGCGTGGCGCTTTCTCATAGCTCACGCTGTAGGTATCTCAGTTCGGTGTAGGTCGTTCGCTCCAAGCTGGGCTGTGTGCACGAACCCCCCGTTCAGCCCGACCGCTGCGCCTTATCCGGTAACTATCGTCTTGAGTCCAACCCGGTAAGACACGACTTATCGCCACTGGCAGCAGCCACTGGTAACAGGATTAGCAGAGCGAGGTATGTAGGCGGTGCTACAGAGTTCTTGAAGTGGTGGCCTAACTACGGCTACACTAGAAGGACAGTATTTGGTATCTGCGCTCTGCTGAAGCCAGTTACCTTCGGAAAAAGAGTTGGTAGCTCTTGATCCGGCAAACAAACCACCGCTGGTAGCGGTGGTTTTTTTGTTTGCAAGCAGCAGATTACGCGCAGAAAAAAAGGATCTCAAGAAGATCCTTTGATCTTTTCTACGGGGTCTGACGCTCAGTGGAACGAAAACTCACGTTAAGGGATTTTGGTCATGAGATTATCAAAAAGGATCTTCACCTAGATCCTTTTAAATTAAAAATGAAGTTTTAAATCAATCTAAAGTATATATGAGTAAACTTGGTCTGACAGTTACCAATGCTTAATCAGTGAGGCACCTATCTCAGCGATCTGTCTATTTCGTTCATCCATAGTTGCCTGACTCCCCGTCGTGTAGATAACTACGATACGGGAGGGCTTACCATCTGGCCCCAGTGCTGCAATGATACCGCGAGACCCACGCTCACCGGCTCCAGATTTATCAGCAATAAACCAGCCAGCCGGAAGGGCCGAGCGCAGAAGTGGTCCTGCAACTTTATCCGCCTCCATCCAGTCTATTAATTGTTGCCGGGAAGCTAGAGTAAGTAGTTCGCCAGTTAATAGTTTGCGCAACGTTGTTGCCATTGCTACAGGCATCGTGGTGTCACGCTCGTCGTTTGGTATGGCTTCATTCAGCTCCGGTTCCCAACGATCAAGGCGAGTTACATGATCCCCCATGTTGTGCAAAAAAGCGGTTAGCTCCTTCGGTCCTCCGATCGTTGTCAGAAGTAAGTTGGCCGCAGTGTTATCACTCATGGTTATGGCAGCACTGCATAATTCTCTTACTGTCATGCCATCCGTAAGATGCTTTTCTGTGACTGGTGAGTACTCAACCAAGTCATTCTGAGAATAGTGTATGCGGCGACCGAGTTGCTCTTGCCCGGCGTCAATACGGGATAATACCGCGCCACATAGCAGAACTTTAAAAGTGCTCATCATTGGAAAACGTTCTTCGGGGCGAAAACTCTCAAGGATCTTACCGCTGTTGAGATCCAGTTCGATGTAACCCACTCGTGCACCCAACTGATCTTCAGCATCTTTTACTTTCACCAGCGTTTCTGGGTGAGCAAAAACAGGAAGGCAAAATGCCGCAAAAAAGGGAATAAGGGCGACACGGAAATGTTGAATACTCATACTCTTCCTTTTTCAATATTATTGAAGCATTTATCAGGGTTATTGTCTCATGAGCGGATACATATTTGAATGTATTTAGAAAAATAAACAAATAGGGGTTCCGCGCACATTTCCCCGAAAAGTGCCACCTAAATTGTAAGCGTTAATATTTTGTTAAAATTCGCGTTAAATTTTTGTTAAATCAGCTCATTTTTTAACCAATAGGCCGAAATCGGCAAAATCCCTTATAAATCAAAAGAATAGACCGAGATAGGGTTGAGTGTTGTTCCAGTTTGGAACAAGAGTCCACTATTAAAGAACGTGGACTCCAACGTCAAAGGGCGAAAAACCGTCTATCAGGGCGATGGCCCACTACGTGAACCATCACCCTAATCAAGTTTTTTGGGGTCGAGGTGCCGTAAAGCACTAAATCGGAACCCTAAAGGGAGCCCCCGATTTAGAGCTTGACGGGGAAAGCCGGCGAACGTGGCGAGAAAGGAAGGGAAGAAAGCGAAAGGAGCGGGCGCTAGGGCGCTGGCAAGTGTAGCGGTCACGCTGCGCGTAACCACCACACCCGCCGCGCTTAATGCGCCGCTACAGGGCGCGTCCCATTCGCCATTCAGGCTGCGCAACTGTTGGGAAGGGCGATCGGTGCGGGCCTCTTCGCTATTACGCCAGCTGGCGAAAGGGGGATGTGCTGCAAGGCGATTAAGTTGGGTAACGCCAGGGTTTTCCCAGTCACGACGTTGTAAAACGACGGCCAGTGAGCGCGCGTAATACGACTCACTATAGGGCGAATTGGAGCTCCAC
